# Targeting severe symptoms of early 2023-new outbreak of COVID-19 by classical drug dexamethasone: a potential fatality reducing agent

**DOI:** 10.1186/s12967-023-04186-4

**Published:** 2023-05-26

**Authors:** Dongsheng Liu, Aamir Saeed, Sidra Majaz, Bashrat Ahmad, Ashfaq Ahmad, Yingqiu Xie

**Affiliations:** 1 Shandong First Medical University, Jinan, China; 2 Department of Bioinformatics, Hazara University, Mansehra, 21300 KPK Pakistan; 3 Department of Biology, School of Sciences and Humanities, Nazarbayev University, Astana, 010000 Kazakhstan

**Dear Editor,**

The Advisory Group on Viral Evolution Technology (TAG-VE) confirmed on January 4, 2023 that 95% of the domestically transmitted case in this round of epidemic in China mainly belong to the Omicron BA. 5.2 or BF. 7 [1]. According to the national coronavirus infection situation released by the China Center for Disease Control and Prevention on January 25, the development of the COVID-19 epidemic shows a trend of first increasing and then decreasing and the number of PCR test positive people reached the daily peak on December 22, 2022 [2].

In this round of epidemic, we can feel that when the medical resources in cities are tight, the rural areas can cope with it with facility. Some think that the smooth pass of the climax of the pandemic in rural areas is attributed to country doctors’ discovering of indigenous methods to effectively deal with COVID-19 infection, which is a “four-piece set of village medicine” consisting of antiviral drugs, antibiotics, hormones and antipyretic drugs. However, in fact, there may be no other choice in rural areas under the shortage of medical supplies and countermeasure. The rural areas are lack of CT and antiviral drugs for COVID-19, and the disease can only be treated by clinical experience and low-cost commonly used drugs. In the “four-piece set of village medicine”, the use of antibiotics and hormones is greatly questioned, among which antibiotics have been shown to be ineffective against COVID-19 or viral infections. Basically, the hormone used by the doctors working in rural areas is dexamethasone, one of the essential drugs in the basic public health system (weixin public account Fenghuoloushi). Dexamethasone is a kind of corticosteroid/immunomodulatory drug recommended by the National Institutes of Health (NIH) in the United States in the COVID-19 Treatment Guidelines, as well as China in Guidelines on the Novel Coronavirus-Infected Pneumonia Diagnosis and Treatment (the 9th/10th Edition) and Guidelines for the diagnosis, treatment and prevention of COVID-19 in adults in China, but it is generally used in the treatment of patient in a critical condition [3]. Dexamethasone has had an immediately effective effect on country doctors’ treating COVID-19 patients. 5 mg dexamethasone sodium phosphate is also injected intramuscularly to infected children with persistent high fever in some urban hospitals, which also has a good antipyretic effect.

## Interaction analyses of SARS-COV-2 RBDs and dexamethasone

We were interested to probe the binding impact of dexamethasone with SARS-CoV-2 Spike RBD and in particular RBD of strain XBB.1.5. Here we considered binding analysis for five strains (Omicron, BJ.1, BM.1.1.1, XBB and XBB) and wild type. The emergence of strain XBB has been reported a recombinant strain BJ.1 and BM.1.1.1, that further evolved to strain XBB.1.5. All these four strains belong to the Omicron lineage which itself is evolved from the wild type strain [3]. To comparatively analyze all these mentioned strains, we used the former five as control.

**Fig. 1:**
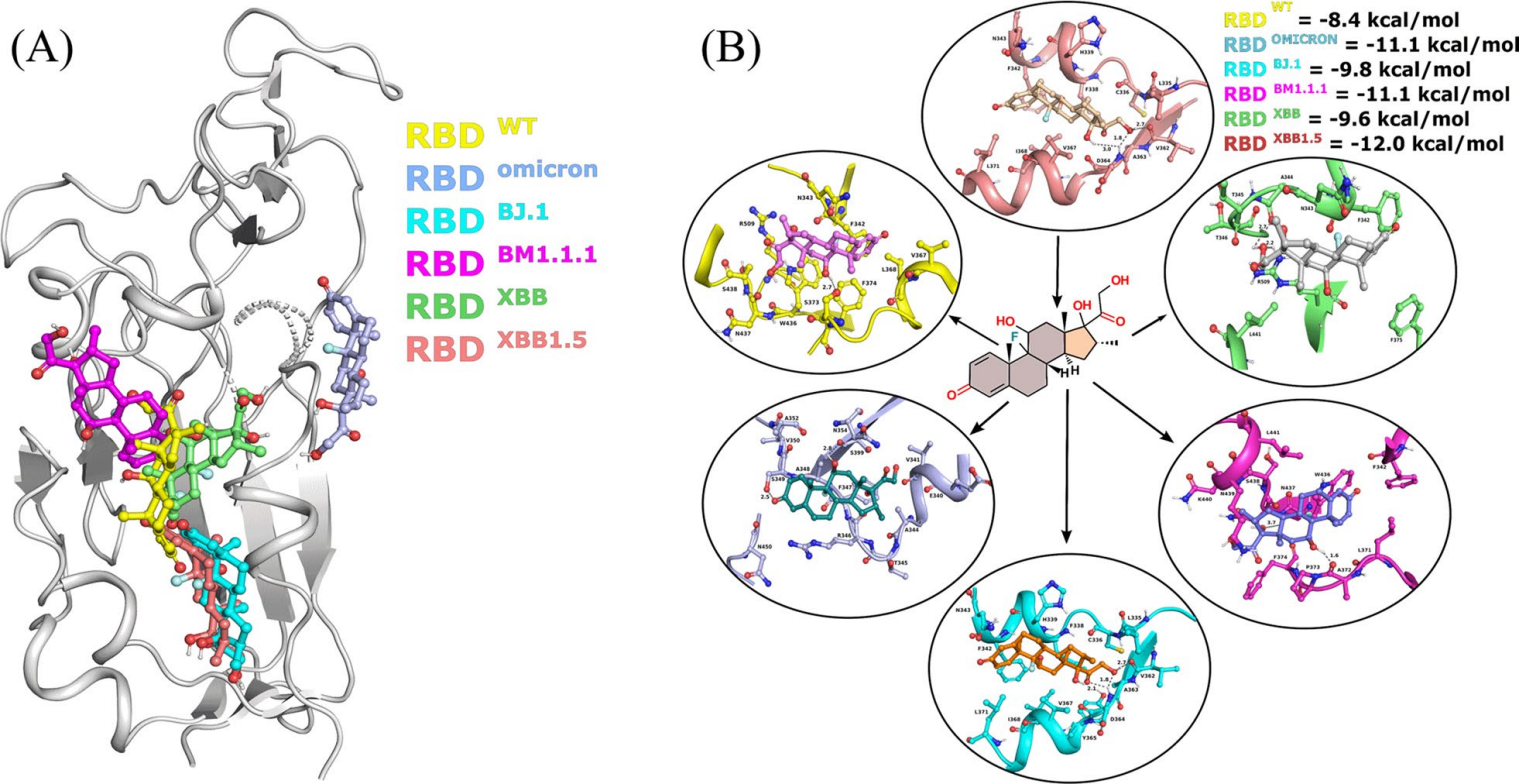
Binding and Energetics Analysis of Dexamethasone against SARS-CoV-2 RBD. **A**, Distribution of dexamethasone binding preferences against the strains of SARS-CoV-2. **B**, Two-dimensional representation of Dexamethasone interaction against the SARS-CoV-2 RBDs of Wild type, variant Omicron, BJ.1, BM.1.1.1, XBB and XBB.1.5, respectively

**Fig. 2:**
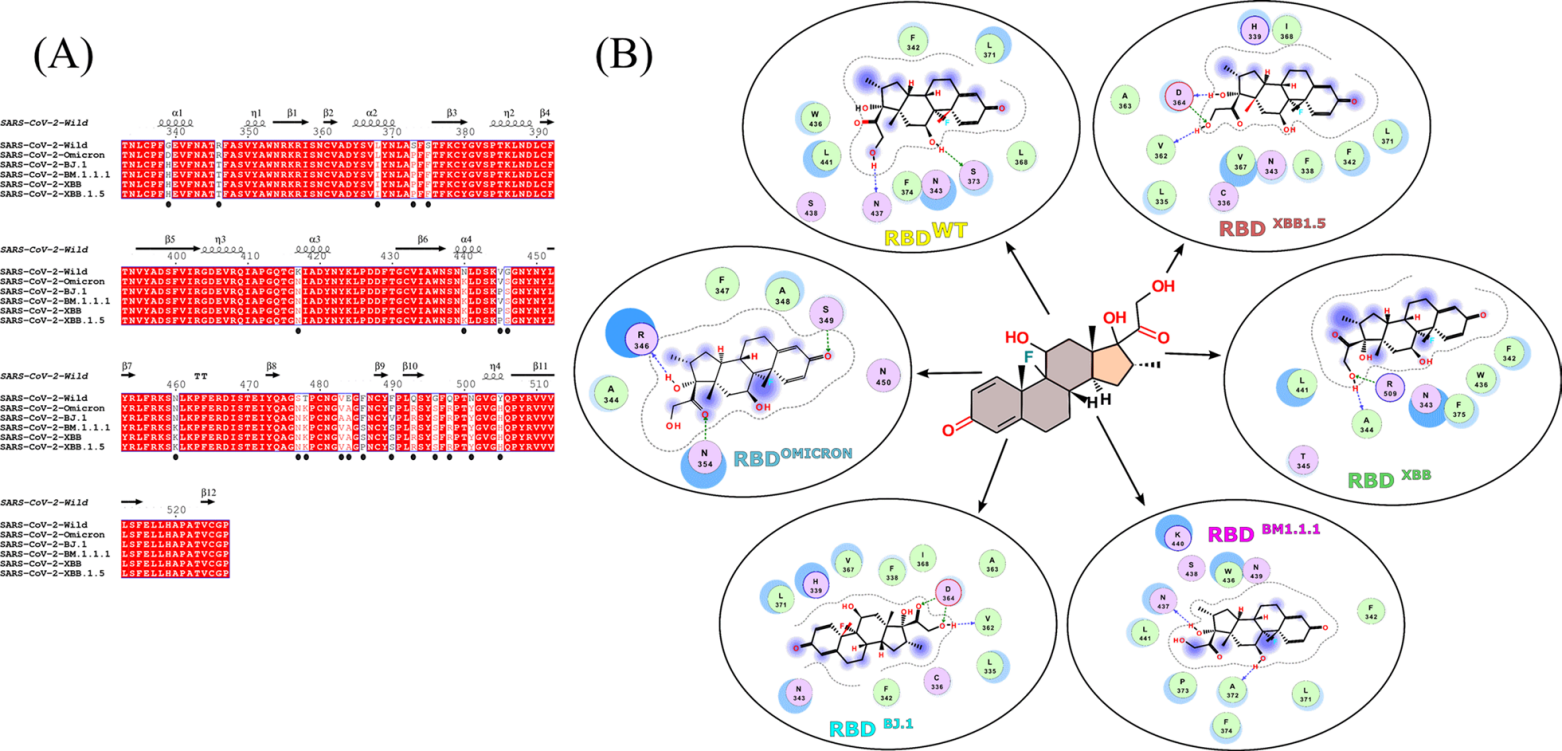
Residue Contributions in Binding of Dexamethasone against SARS-CoV-2 RBD. **A**, SARS RBD differences at amino acid level among different strains. **B**, Details of the binding pocket and residue of the SARS-CoV-2 RBDs of Wild type, variant Omicron, BJ.1, BM.1.1.1, XBB and XBB.1.5, for Dexamethasone

Dexamethasone targeted the middle pocket enclosed by the two smaller α helices located below the ACE2 binding interface, and showed binding energies of -8.4, -11.1, -9.8, -11.1, -9.6 and -12.0 Kcalmol^−1^ with RBD wild type, Omicron, BJ.1, BM.1.1.1, XBB and XBB.1.5, respectively. However, dexamethasone did not prefer the same pocket and docked to the dorsal side of pocket in Omicron variant (Fig. 1). To confirm any aberrant changes in RBD static structures brought by mutations, all the structures were aligned and found satisfactory, with RMSD of 0.2–0.5 Å suggesting no major structural aberrations (Additional file 1: Figure S1). One of the apparent reasons we found was 339 mutation (a pocket residue), which was an opposite charged residue in Omicron lineages compared to other members and Glycine in wild type. Aside binding conformations, we observed two hydrogen bonds as well as vdw interactions with S373 and W436 in wild type. In contrast, the Spike proteins of BJ.1 and BM.1.1.1 variant displayed three and two hydrogen bonds with V362, D364 and A372, N437 assisted by vdW effects. With the Spike RBD of strain XBB, dexamethasone was able to form hydrogen bond interaction with A344, T346 and R506 along with N343, F342, T345, F375, W436 and L441 observed in vdW interactions. The RBD of strain XBB.1.5, engaged the polar oxygen of dexamethasone and formed two hydrogen bonds with D364 and V362. Apart from hydrogen bonds, we found residues L335, C336, F338, H339, F342, N343, A363, V367, I368 and L371 involved in vdW interactions (Fig. 2).

Dexamethasone is a powerful immunosuppressive agent with powerful anti-inflammatory effect, which can fundamentally inhibit the inflammatory response of the body against viral infection, reduce endogenous pyrogen, and inhibit the response of the hypothalamus, with obvious cooling and antipyretic effect. However, dexamethasone, as a hormone drug, has certain side effects and harm, especially for children and people with pre-existing conditions. Therefore, dexamethasone can be used as a temporary antipyretic drug under strict control of dosage when meeting an urgent need, but it cannot be used as a conventional therapy for patients with mild symptoms. The long term use of it will cause a series of side effects, such as hyperglycemia, insulin resistance, lipid metabolism disorder, muscular dystrophy, osteoporosis, etc. Studies have confirmed that physical exercise can improve the side effects caused by long-term use of dexamethasone [4, 5]. For example, low-intensity exercise can improve hyperglycemia and gastric dysmotility and Moderate -intensity aerobic exercise and high-intensity interval exercise can improve insulin resistance and lipid metabolism disorders [4, 5]. Therefore, moderate physical exercise is recommended after treatment with dexamethasone to reduce the side effects caused by it.

## Supplementary Information

**Additional file 1: Figure S1.** RMSD Analysis among the predicted structure.

## Data Availability

Analyzed data can be found from supplementary data or by request.
